# *mcr-1* Gene in Latin America: How Is It Disseminated Among Humans, Animals, and the Environment?

**DOI:** 10.3389/fpubh.2021.648940

**Published:** 2021-05-07

**Authors:** Silvia Adriana Mayer Lentz, Tanise Vendruscolo Dalmolin, Afonso Lus Barth, Andreza Francisco Martins

**Affiliations:** ^1^Programa de Ps Graduao em Microbiologia Agrcola e Do Ambiente, Universidade Federal Do Rio Grande Do Sul, Porto Alegre, Brazil; ^2^Laboratrio de Microbiologia Aplicada, Instituto de Cincias Bsicas da Sade, Universidade Federal Do Rio Grande Do Sul, Porto Alegre, Brazil; ^3^Faculdade de Sade, Departamento de Farmcia, Universidade de Braslia (UnB), Braslia, Brazil; ^4^Laboratrio de Pesquisa em Resistncia Bacteriana (LABRESIS), Hospital de Clnicas de Porto Alegre, Porto Alegre, Brazil

**Keywords:** *mcr-1* gene, IncX4 plasmid type, colistin resistance, Latin America, antimicrobial resistance

## Introduction

In the last decade, polymyxins have been reintroduced in the therapeutic arsenal to treat severe infections by carbapenem-resistant Enterobacterales. At that time, reports of polymyxin resistance were all due to chromosomal mutations (1). These mechanisms included (i) modifications of the lipopolysaccharides (LPSs) moiety via the addition of cationic groups; (ii) mutations that lead to the loss of the LPS; (iii) porin mutations and overexpression of efflux pump systems; (iv) overproduction of capsular polysaccharide (CPS) in some Gram-negative bacteria (GNB) that hide the polymyxin-binding sites and the release of CPS-trapping polymyxins; and (v) enzymatic inactivation of polymyxins (2). Although some chromosomal resistance mechanisms have been studied since the 1960's, it was in the late 1990's, after the reintroduction of polymyxins in the therapeutic arsenal, that this problem became more important (3). In fact, this information is supported by the first report of colistin resistance among *Acinetobacter baumannii* clinical isolates from the Czech Republic in 1999 and *Klebsiella pneumoniae* from Athens in 2004 (4).

However, in 2015, the *mcr-1* gene, associated with IncI2-type plasmid, was identified in *Escherichia coli* resistant to colistin obtained from food animals and humans in China (1). This finding promoted a great concern in the international scientific community since the last therapeutic option to treat serious infections by multidrug-resistant GNB could be exhausted. With the horizontal transfer, the rapid spread of the *mcr-1* gene would be inevitable.

The *mcr-1* gene carried by different plasmid types has already been identified in all five continents from different sources and hosts (1, 5). Surprisingly, Shen and colleagues, in a retrospective study, characterized the early occurrence of the *mcr-1* gene in chicken isolates from 1980's (6).

So far, a total of 10 different variants (7) of the *mcr* gene have been described mainly among the Enterobacterales, but with the *mcr-1* gene remaining the most prevalent (1). To date, the sequences of 30 *mcr-1* mutations (*mcr-1.2* to *mcr-1.30*) have already been deposited in the GenBank database, differing from *mcr-1* by one or few amino acids. Besides that, 10 *mcr* gene variants (*mcr-1* to *mcr-10*) were deposited, with amino acid identity ranging from 31 to 83% (8). These variants were identified at the beginning in Enterobacterales isolates, including *E. coli* (*mcr-1, mcr-2*, and *mcr-3* genes), *Salmonella enterica* (*mcr-4, mcr-5* and *mcr-9* genes), *K. pneumoniae* (*mcr-7* and *mcr-8* genes), and *Enterobacter roggenkampii* (*mcr-10* gene). The exception is due to *mcr-6* gene that was first identified in *Moraxella* spp. After that, some variants were identified in non-fermenter Gram-negative rods, as *Acinetobacter* spp. (*mcr-1* and *mcr-4*) and *Pseudomonas* spp. (*mcr-1* only) (9, 10).

In general, the isolates carrying *mcr* genes were first isolated from animals such as pigs (*mcr-1, mcr-2, mcr-3, mcr-4, mcr-6*, and *mcr-8* genes) and chickens (*mcr-5* and *mcr-7* genes), but *mcr-9* and *mcr-10* genes were identified, for the first time, from human patients (8).

## Epidemiology Of Polymyxin Resistance

The resistance to polymyxins was attributed mainly to chromosomal mutations and is rare in human clinical isolates (0.671.6%) (11). Nevertheless, this differs among bacteria species, being higher in *K. pneumoniae* and *A. baumannii* (2080%) (4) in contrast to lower rates in *E. coli* (0.20.6%) (11).

The polymyxin resistance rate associated to plasmid, as *mcr-1*, is also low in humans (~1%) (4). On the other hand, according to a large US surveillance study, the association between *mcr-1* and other antibiotic resistance genes, such as extended-spectrum -lactamase (ESBL) and carbapenemases, may reach 32% of prevalence in *K. pneumoniae* (11). Regarding the mortality associated with infections caused by colistin-resistant isolates in humans, the rate is variable, and it is higher in critically ill patients (3037%) including those previously exposed to colistin (4). The mortality rate may reach 100% in patients with nosocomial infections caused by pandrug-resistant *K. pneumoniae*.

It is important to emphasize that the prevalence of *mcr-1* gene is higher among production animals, mainly in pig and chicken isolates (5). The data show colistin resistance rates of ~70% in *E. coli* isolates from China and ~90% among Enterobacterales in some European countries (8). So, these data corroborate with the scientific evidence that the worldwide spread of the *mcr-1* gene is mainly associated with the large amounts of colistin use in production animals, and its emergence is a particular threat to public health as colistin is considered the last-resort antimicrobial for treatment of severe human infections, and its use in livestock production contributes to emerging resistance globally (1).

## *mcr-1* In Latin America

In Latin America, a systematic review analysis showed that the prevalence of *mcr-1* gene is higher in isolates from animals (8.7%) than in food (5.4%) and humans (2.0%) (12). To the best of our knowledge, the first reports of *mcr-1* gene in Latin America dated from July and October 2012 when this gene was identified in *E. coli* isolates from two inpatients in different hospitals in Argentina (Table 1) (13). Patients presented neurological disease and diabetes, and the *mcr-1*positive isolates were obtained from blood and urine, respectively. In this study, the authors evaluated the presence of the *mcr-1* gene in 87 colistin-resistant clinical human isolates from 2008 to 2016 (28 *E. coli*, 19 *K. pneumoniae*, 36 of other members of the Enterobacterales, and 4 non-fermenter Gram-negative rods), and nine isolates of *E. coli* were *mcr-1* positive. These isolates were associated with human infections, mainly in males, and the average age of the patients was 68.5 years. All *mcr-1*positive *E. coli* isolates were genetically unrelated as determined by pulsed-field gel electrophoresis, and the resistance mechanism was horizontally transferable by conjugation (13). Still, in 2012, other studies reported *mcr-1* harboring *E. coli* recovered from Kelp Guls in Argentina (14) and from swine in Brazil (Table 1) (15).

**Table 1:** Summary of mainly studies reporting *mcr-1* gene in Latin America.

**Period of the study**	**Country**	**Source of Isolate**	**Total Isolates (*mcr*-carried)**	**Species**	**Plasmid Type**	**Sequence Type (ST)**	**Genetic Context**	**References**
20002016	Brazil	Fecal samples-chicken and swine (Production Animals)	515 (16)	*E. coli*				(15)
20022016	Colombia	Urine vaginal secretion blood stool tissue right toe leg secretion abdomen abscess (Human)	513 (12)	*E. coli* *S. enterica* Typhimurium *K. pneumoniae*	IncP-1 IncFII IncHI1 IncH	*E. coli* (ST10, ST37, ST101, ST744, ST1263, ST3056, and ST6627) *S*. Typhimurium (ST34) *K. pneumoniae* (ST307)	IS*Apl1-mcr-1-pap2* (IncP-1) *mcr-1-pap2* (IncP-1)	(17)
20082016	Argentina	Urine, blood, abdomen, abscess, bone (Human)	87 (9)	*E. coli*				(13)
2012	Argentina	Fecal samples - Kelp gulls penguin (Wild Animal)	50 (5)	*E. coli*	IncI2	ST101 and ST744	IS*Apl1*-*mcr-1*	(14)
20122018	Argentina	Urine, blood, other samples (Human)	192 (192)	*E. coli*	IncHI2 IncX4	ST10, ST156, ST354, ST8492, ST5208		(37)
2013	Bolivia	Potatoes (Food)	83 (1)	*C. braakii*	IncI2			(16)
2013	Argentina	Fecal samplesChicken (Production Animals)	10 (10)	*E. coli*	IncI2	ST155 (CC10: ST10, ST1141 and ST1286), ST617, ST10, ST410, ST1011, ST1408	IS*ApI1-mcr-1.5-pap2*- IS*ApI1*	(33)
20132014	Ecuador	Feceschicken (Production Animals)	176 (6)	*E. coli*				(24)
20132016	Brazil	Meat Poultry (Food)	60 (2)	*Salmonella enterica* serovar Schwarzengrund	IncX4	ST96	*parA* and hypothetical protein upstream *mcr-1* and *pap2* downstream	(44)
20132017	Chile	Urine (Human)	13 (1)	*E. coli*	IncI2	ST4204 (CC10)	*mcr-1* was delimited upstream by a gene that encodes a *pap2* protein and downstream by a relaxase-encoding gene (*nikB*)	(18)
2014	Argentina	Clinical samples - dogs and cats (Pets)	54 (1)	*E. coli*	IncI2	ST770	*mcr-1* was delimited upstream by *nikB* gene which encodes a relaxase and *pap2* downstream	(31)
20142017	Brazil	Pork carcasses (Food)	490 (8)	*S. enterica* serovar Typhimurium	IncX4	ST19 ST4556 ST50	*mcr-1* was delimited upstream by IS*26* and hypothetical protein and *pap2* downstream	(26)
2015	Venezuela	Fecal samples (Human and Animal)	93 (2)	*E. coli*	IncI2	ST452 and ST19	Absence of IS*Apl1*	(23)
2015	Mexico	Swine stool samples (Production Animal)	1 (1)	*E. coli*	Incp0111	ST744	IS*Apl1* upstream *mcr-1* gene	(22)
20152016	Brazil	Rectal swab and urine (Human)	140 (2)	*E. coli*	IncX4	ST206 and ST354	*mcr-1* was delimited upstream by IS*26* and hypothetical protein and *pap2* downstream	(46)
2016	Brazil	Seawater (Environment)	11 (3)	*E. coli*	IncX4			(36)
2016	Ecuador	Fecal swabs and soil fecal from chicken and two dogs (Domestic Animals)	42 (3)	*E. coli*	IncI2	ST3941, ST1630, ST2170	*mcr-1* was delimited upstream by *nikB* gene and *pap2* downstream	(32)
2016	Brazil	Rectal swab (Human)	3 (3)	*E. coli* and *K. pneumoniae*	IncX4	*E. coli* ST744 and *K. pneumoniae* ST101		(38)
2016	Bolivia	Fecal samples (Human)	337 (173)	*E. coli, C. europaeus, E. hormaechei*	IncI2 and IncHI1 (*E. coli*); *Citrobacter* and *Enterobacter (*IncI2)	*E. coli* (ST48, ST744, ST10, ST206, ST2705, ST2936, ST1286, ST7,570, ST69, ST10, ST117, ST711, ST7571, ST3056)	*mcr-1*-*pap* (IncI2) *mcr-1.5*-*pap* IS*Apl1* (IncHI1) IS*Apl1-mcr*-*1*-*pap*-IS*Apl1* (IncHI1)	(27)
20162017	Paraguay	Urine and feces (Human)	150 (7)	*K. pneumoniae, E. coli*, and *S*. Schwarzengrund				(20)
2017	Brazil	Water Sample from a mangrove (Environment)	1 (1)	*E. coli*	IncX4			(39)
2017	Uruguay	Blood, rectal swab, and urine (Human)	3 (3)	*E. coli*	IncI2 e IncX4	ST10, ST93, and ST5442		(19)
2017	Peru	Urine (Human)	10 (7)	*E. coli*				(21)
2019	Brazil	Fecal sample and Water from Zoo (Wild Animal and Environment)	27 (5)					(28)
2020	Brazil	Blood, urine, and peritoneal fluid (Human)	100 (2)	*E. coli* and *K. pneumoniae*	IncX4	ST471/ST410 (*E. coli)* and ST15 (*K. pneumoniae*)		(29)

*: No data*.

Since 2012, the *mcr-1* gene has already been identified in bacteria from humans, animals, animal food products, and environmental sources in different countries in Latin America, including Brazil (15), Bolivia (16), Colombia (17), Chile (18), Uruguay (19), Paraguay (20), Peru (21), Mexico (22), Venezuela (23), and Ecuador (24). Brazil is the country with the highest number of *mcr-1*positive bacteria reported in Latin America mainly from bacterial isolates obtained from poultry rectal swabs (15) (Table 1).

It is important to consider that Brazil is the fourth largest pork producer and exporter and the largest chicken meat exporter in the world, which could contribute to the high prevalence of the *mcr-1* gene in this country (25). As in other countries, the colistin was extensively used in Brazil as a growth promoter for many years. In 2016, the government published restrictions on the use of colistin in animal production (1, 26), which came into force in 2018. However, the use of colistin to treat or prevent infections in veterinary medicine including animal productions is still allowed.

*E. coli* is the most common species harboring the *mcr*-*1* gene in Latin America countries. However, many other Enterobacterales members such as *K. pneumoniae, Salmonella* spp., *Citrobacter* spp., and *Enterobacter* spp. were also reported as positive for the *mcr*-*1* gene (17, 27). In addition to *mcr-1*, other variants of the gene were reported rarely in Latin America, such as *mcr-3, mcr-5, mcr-7*, and *mcr-9* (28,30).

## Genetic Context And Dissemination Of *mcr-1* GENE

*E. coli* isolates harboring *mcr-1* gene belong to different sequence types (STs) (31, 32) (Table 1), indicating that the dissemination of the *mcr-1* gene is associated with different clonal strains (1). Loayza-Villa and colleagues investigated the relationship between an *E. coli* carrying *mcr-1* recovered from the gastrointestinal tract of a boy and an *mcr-1*positive *E. coli* from fecal samples and rectal/cloacal swabs from his domestic animals. *E. coli* strains from domestic animals and from the boy were different; however, all plasmids harboring the *mcr-1* gene shared 90% nucleotide identity and a highly conserved backbone, supporting the idea of horizontal dissemination of the *mcr-1* gene (32).

In Latin America, the *E. coli* belonging to CC10 clonal complex, known as the largest human clonal complex, was the most reported in previous studies, including the ST744 and ST10 (1, 17, 22, 33). *E. coli* CC10 strains are widely disseminated among humans, animals, meat products, and environmental sources (34, 35) and are designated as multidrug-resistant strains carrying frequently ESBL, among others (5, 31).

The *mcr-1* gene is carried by a wide range of conjugative and non-conjugative plasmid types, including IncX3, IncX4, an IncX3X4 hybrid, IncH1, IncHI1, IncHI2, IncP, IncI2, IncF, IncFII, an IncI2IncFIB hybrid, and IncY (5). The *mcr-1* gene can also be integrated into the chromosome of some strains (17). However, in Latin America, only four plasmids have been described so far: IncX4 (36), IncP (22), IncI2 (31), and IncHI2 (37), of which the IncX4 plasmid is the most frequent in Brazil (38, 39) (Table 1). There is a clear association between the IncX4 plasmids and the insertion sequences associated with the dissemination of the *mcr-1* gene (40).

Plasmid analysis has revealed that the insertion sequence IS*Apl1* (which belongs to the IS30 family transposase), in a composite transposon (IS*Apl1*-*mcr-1*-IS*Apl1*), is usually present in IncHI2-type plasmids (size of 200 kb), being either present or absent in IncI2-type plasmids (60 kb), and completely absent in IncX4-type plasmids (30 kb) (Table 1).

The role of IS*Apl1* in the mobilization of the *mcr-1* gene was demonstrated *in vitro* by transposition. It was suggested that the recombination events associated with mobilization of the *mcr-1* gene were initially mediated by two copies of IS*Apl1* from an unknown progenitor to a plasmid and subsequently transferred to Enterobacterales (41).

Besides that, according to Snesrud et al., the presence of a single or two copies of IS*Apl1* indicates a recent acquisition of the *mcr-1* gene, whereas the absence of this insertion sequence could be correlated with the adaptation of the *mcr-1* gene to a new host (41).

The regulation mechanism of *mcr-1* gene expression is complex and remains unknown. In general, the gene expression is controlled by its promoter and the corresponding activators and/or inhibitors. Zhang et al. suspect that genes encoding activators and/or inhibitors in the host chromosome may affect the expression of the *mcr-1* gene found on plasmids IncX4 and other plasmids. They may vary expressively in unlike genetic backgrounds of the different strains and/or *mcr-1*harboring plasmids, despite that their promoters are remarkably similar (42).

Although the mobility and dissemination of the *mcr-1* gene are associated with IS*Apl1* and the *pap2* gene in most plasmid types (43), the genetic context of the IncX4 plasmid type, in Latin America, is different. This context is characterized by lacking the IS*Apl1*, but it preserves the *pap2* sequence and a hypothetical protein (hp) around the *mcr-1* gene (26, 44). What would be the explanation for that?

Snesrud et al. analyzed the genetic environment of the *mcr-1* gene associated or not with IS*Apl1* and concluded that the target site duplications generated by IS*Apl1* transposition are present even in lack of the IS*Apl1*. This result suggests that the mechanism to mobilize the *mcr-1* gene is the same as that observed in other plasmids, and after that, the loss of the insertion sequence by recombination events in IncX4 occurs (45).

Furthermore, the IS26 mobile element upstream to the *mcr-1* gene has been also associated with IncX4 plasmid types in Brazil, but there are no other reports in Latin America (26, 46) (Table 1). This Insertion Sequence (IS) plays an important role in the dissemination and evolution of the antimicrobial resistance genes on plasmids, including colistin resistance genes (1).

## Discussion

In veterinary medicine, colistin is mainly administrated in pigs and poultry production, for prophylaxis or treatment. The spread of colistin resistance may lead to treatment failure, as well as increase the pathogen transmission reach with quality and economic loss in production animals.

Strong scientific evidence indicates that the *mcr-1* gene might have originated from animals because (i) colistin has been used extensively for decades in veterinary practices; (ii) *mcr-1* gene was largely identified in several animals and animal food products; (iii) the identification of the *mcr-1* gene in *E. coli* isolate recovered before 1980 in China suggests that the emergence of this gene may be linked to the use of colistin as a growth promoter in the poultry industry; and (iv) genetic features of *mcr-1* gene associated with IS*Apl1* were first identified in *Actinobacillus pleuropneumoniae*, a common animal pathogen (43), which could be involved in recombination events leading to the mobilization of the *mcr-1* cassette.

Finally, a recent study has demonstrated that when colistin is banned from use in animal feed, there was a significant decrease of the *mcr-1* gene prevalence in most sources, including pig farms, food, and environment samples (47). Given that the production animals can be a reservoir for *mcr-1* gene and its dissemination can occur by food and environment, all countries should apply surveillance, monitoring, and restrictive measures to polymyxins use. In Latin America, Brazil, and Argentina (1) have already banned the use of colistin as a growth promoter, but the impact of this measure has not been evaluated yet.

The problem of antimicrobial resistance is related to the use and abuse of antibiotics in humans, animals, and the environment. Besides that, the *mcr-1* gene is disseminated mainly by *E. coli* clones, with a high capacity to survive in different ecological niches, some of them with pandemic and epidemic potential. So, it seems clear that the One Health approach should be adopted to integrate veterinary and human medicine to address antimicrobial resistance.

## Author Contributions

SAML, TVD, and AFM: conception of the opinion, collected data, and wrote the paper. ALB and AFM: reviewed and edited. All authors contributed to the article and approved the submitted version.

## Conflict of Interest

The authors declare that the research was conducted in the absence of any commercial or financial relationships that could be construed as a potential conflict of interest.
